# Electrical vestibular stimulation to improve balance in older adults: a pilot randomized controlled trial

**DOI:** 10.1186/s12984-025-01749-y

**Published:** 2025-10-31

**Authors:** Jordan A. King, Noah Walters, Nadine Rodrigues, Jenna Al Bastami, Niki Mehri, Adrian Chan, Madison Spencer, Sadie Clark, Evan Ferrier, Serena L. Orr, Jocelyn Rempel, Andreas Hauenstein, Joshua M. Roper, John D. Ralston, Ryan M. Peters

**Affiliations:** 1 Department of Biomedical Engineering, University of Calgary, Calgary, AB Canada; 2 Hotchkiss Brain Institute, Calgary, AB Canada; 3 Faculty of Kinesiology, University of Calgary, Calgary, AB Canada; 4 Department of Pediatrics, Cumming School of Medicine, University of Calgary, Calgary, AB Canada; 5 Department of Community Health Sciences, Cumming School of Medicine, University of Calgary, Calgary, AB Canada; 6 Department of Clinical Neurosciences, Cumming School of Medicine, University of Calgary, Calgary, AB Canada; 7 Alberta Children’s Hospital Research Institute, Calgary, AB Canada; 8 Centre for Health and Innovation in Aging, Mount Royal University, Calgary, AB Canada; 9 Neursantys, Inc., Menlo Park, CA USA

**Keywords:** Vestibular system, Balance control, Aging, Electrical vestibular stimulation, Galvanic vestibular stimulation, Postural stability, Phybrata power, Vestibular therapy, Balance therapy

## Abstract

**Background:**

Falls are the leading cause of injury-related hospitalizations among older adults, often linked to vestibular dysfunction. While vestibular rehabilitation therapy is a standard intervention, designed to compensate for vestibular impairment with proprioceptive and visual cues, potential cumulative effects of noisy Electrical Vestibular Stimulation (nEVS) on balance improvement in older adults are not well understood.

**Objective:**

This study evaluated the efficacy of cumulative nEVS dosing in improving static balance, its potential mechanisms, and clinical significance.

**Methods:**

A single-blind, pilot randomized controlled trial enrolled 40 older adults (mean age: 77.7 ± 11.8 years). Participants were randomly assigned to a Stimulation group (nEVS intervention) or Sham group. The nEVS regimen included low-amplitude wideband stimulation (± 0.35 mA, 0.001–300 Hz) for 20 min, three times weekly for six weeks. Balance performance was assessed immediately before and after nEVS using a head-mounted sensor to measure physiological vibration acceleration (‘Phybrata’) power as a measure of postural stability in four conditions: Floor Eyes Open, Floor Eyes Closed, Foam Eyes Open, and Foam Eyes Closed. Follow-ups occurred at 3 months and 6 months post-intervention.

**Results:**

The Stimulation group exhibited significant and sustained reductions in Phybrata power with improvements observed as early as Session 3 and persisting through 6 months in Foam EC. Additionally, the Sham group demonstrated smaller reductions in Phybrata power, potentially reflecting a learning effect.

**Conclusion:**

nEVS may be a safe and effective intervention for improving balance in older adults. Its benefits in addressing age-related deficits in balance and sensory integration highlight its potential for fall prevention and rehabilitation. This study was retrospectively registered as a clinical trial on February 25, 2025 (NCT06846047).

**Supplementary Information:**

The online version contains supplementary material available at 10.1186/s12984-025-01749-y.

## Introduction

Every year, 20 to 30% of older Canadians report experiencing a fall, making falls the leading cause of injury-related hospitalizations in this demographic [1]. This incidence rises to 32 to 42% among those aged 70 and older [2]. Prior research has identified dizziness and imbalance as prevalent in up to 30% of older adults, with the risk of falls increasing significantly after the age of 60 [3, 4]. Falls cost the Canadian healthcare system ~$10.3 billion per year [5]. Injury due to falls can negatively impact the quality of life for older people and can lead to social isolation, loss of autonomy, bone fractures, reduction in mobility, and increased risk of osteoarthritis. As the global population aged 60 and over is projected to increase from 12 to 22% by 2050, the search for effective treatments for balance deficits associated with aging becomes increasingly urgent [6]. Currently, vestibular rehabilitation therapy (VRT) is the standard of care for nearly all vestibular injuries, excluding unstable lesions and ongoing labyrinthine pathology [7]. VRT is an exercise-based regimen typically delivered by physical and occupational therapists who have specialized training. The mechanisms behind VRT include vestibular adaptation, which relies on readjusting the gain of vestibular reflexes, and vestibular substitution, which uses alternative sensory strategies (visual and proprioceptive) to replace vestibular function [7].

Human static and dynamic balance depends on central nervous system (CNS) integration and processing of somatosensory, visual, and vestibular sensory inputs to generate motor control outputs [3, 8]. Within the vestibular system, there are the otolith organs and semicircular canals which detect linear and rotational motion of the head, respectively, and provide this sensory information to the vestibular nuclei via the vestibulocochlear nerve. From there, vestibular information relays to ocular motor nuclei to stabilize gaze, the neck and body to stabilize posture, and the cortex for conscious perception of body motion and orientation [8]. These pathways within the vestibular system collectively manage static and dynamic balance, gait, and posture in humans [9, 10].

Vestibular dysfunction is common among older populations [3, 8]. Age-related degeneration, also known as presbyvestibulopathy, affects nearly every type of vestibular-related cell and neural connection, including sensory end-organ hair cells, afferent nerve fibers, scarpa ganglion cells, vestibular nucleus neurons, and Purkinje cells within the cerebellum [8, 10, 11]. Presbyvestibulopathy has a variable etiology profile in the population of older adults, and can result in different patterns of impairment, from peripheral neurosensory loss to central involvement [11]. This general loss of input and central integration of vestibular signals results in increased balance deficits with aging. Multiple pathophysiological mechanisms have been proposed to explain vestibular dysfunction in aging. The most common cause of balance dysfunction is the loss of mechanosensory hair cells [12]. However, given the poor correlation between clinical and histological findings, it is suggested that the vestibular system has compensatory mechanisms that can mask the effects of degeneration [8]. One example of a compensatory mechanism is central gain enhancement, where vestibular processing is amplified within the central nervous system (CNS) [13–15]. Other hypotheses suggest neuroplasticity in the central vestibular pathways [13]. Balance and gait stability begin to deteriorate once peripheral and central vestibular deficits surpass the compensatory capacity of the system [13, 16].

A growing body of research suggests that noisy Electrical Vestibular Stimulation (nEVS) is a promising approach to improving age-related declines in vestibular function [17–21]. nEVS aims to enhance vestibular function by delivering imperceptible electrical currents to vestibular end organs and afferent neurons via electrodes placed over the left and right mastoids [19]. One hypothesized benefit of nEVS is that low levels of noise applied to the vestibular system can enhance the detection of sub-threshold signals during stimulation [19]. This mechanism during stimulation is believed to involve stochastic resonance, where the addition of noise enhances the detection of a signal that would otherwise be undetectable [19]. EVS stimulates hair cells and the vestibulocochlear nerve (CN VIII) evoking vestibular reflexes [22, 23]. nEVS has the potential to be a feasible, safe, and non-invasive treatment for balance deficits. Studies have identified the clinical applications of nEVS in treating conditions such as Meniere’s disease, vestibular neuritis, bilateral vestibular disorders, vestibular schwannoma, Parkinson’s disease, central ischemic lesions, motor myelopathies, anxiety, cognitive disorders, memory disorders, and age-related imbalance [24].

Few studies have examined the effects of nEVS on older populations and only one previous study has investigated the cumulative impact of repetitive nEVS on an elderly cohort [25]. Although this latter study combined nEVS with a 6-week exercise-based treatment program, it did demonstrate a significant improvement in balance and reduction in fall risk in both the exercise-only and exercise with nEVS groups, and the treatment benefits were significantly larger when nEVS was included. Two previous studies of nEVS stimulation in older adults demonstrated reductions in center of pressure and sway length measured using force plates following a single treatment session, indicating improvement in postural stability which persisted for several hours after stimulation [26, 27]. It is unknown if repeated nEVS sessions alone induce greater and sustained improvements in postural stability in healthy adults and aging populations [26]. However, as indicated in a recent review article, the effectiveness of EVS is variable, and not all choices of stimulation parameters are effective for improving postural stability [28]. Emerging evidence suggests that EVS may enhance the outcome of VRT and other exercise-based balance therapies when used in tandem, particularly in individuals with vestibular dysfunction, by accelerating vestibular compensation and reinforcing sensorimotor adaptation mechanisms [25, 29, 30]. nEVS-induced changes in balance could result from improved vestibular information processing in vestibular afferents or the activation of cortical regions, but the precise mechanism remains unknown [19]. There is also no consensus on optimal EVS delivery protocols [19, 28].

This study aims to advance and build upon existing research by developing a fixed regimen of repeated nEVS dosing sessions designed to maintain and restore vestibular system function and central processing to improve balance performance. The nEVS regimen delivered low-amplitude wideband stimulation at ± 0.35 mA from 0.001 to 300 Hz for 20 min, three times a week, over six weeks. Our central hypothesis is that repeated exposure to nEVS will drive sustained neuroplastic improvements in vestibular function that accumulate over a 6-week stimulation protocol, leading to measurable improvements in postural stability that persist for six months or longer post-stimulation.

## Methods

### Participants

Inclusion criteria required participants to be adults aged 50+, as vestibular dysfunction has been identified as the primary cause of balance decline in more than 55% of adults over age 50 [3, 31–33]. Exclusion criteria included individuals with a hearing aid, pacemaker, neurological disorder, or musculoskeletal disorder that significantly impaired balance or mobility. Participants with musculoskeletal conditions were eligible unless those conditions caused observable impairments in mobility or balance (e.g., severe joint instability or pain). While individuals with joint replacements or age-related osteoarthritis may have participated, those with mobility compromising symptoms were excluded. Participants were also required to have normal or corrected-to-normal vision. Specific neurological diagnoses excluded from participation were diabetic neuropathy, idiopathic peripheral neuropathies, Charcot–Marie–Tooth disease, Guillan–Barre syndrome, Alzheimer’s disease and other dementias, cerebellar degeneration/ataxia, brain tumors, traumatic brain injury with residual deficits, essential tremor with gait disturbances, stroke, Parkinson’s disease, Huntington’s disease, multiple sclerosis, chronic migraines, and vestibular disorders such as benign paroxysmal positional vertigo, vestibular neuritis, Meniere’s disease, and bilateral vestibular hypofunction. Participants were screened based on self-report since we did not have access to previous medical records. Data was gathered from retirement and assisted living communities (*n* = 21) and in-lab (*n* = 19) using the same protocol. All testing, regardless of site, was performed in a quiet room, the lighting and flooring were similar, and the instructions given to participants were identical and delivered from the same experimenters across sites. All procedures were approved by the University of Calgary Conjoint Health Research Ethics Board (Ethics ID: REB22-1006) prior to participant enrollment and retrospectively registered as a clinical trial on February 25, 2025 (NCT06846047). All participants provided written informed consent before participation. Data collection ceased after recruiting 48 participants, with final analyses completed on participants that completed all testing sessions (*n* = 40).

### Experimental design

**Fig. 1:**
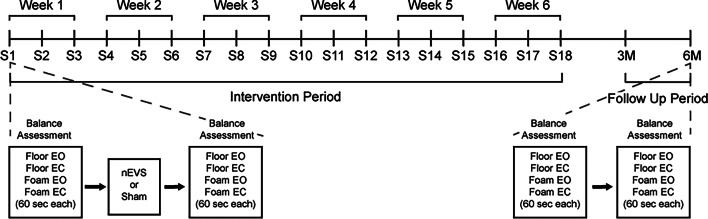
The study consisted of 18 sessions across a 6-week intervention period (S1–S18), followed by two follow-up assessments at 3 months (3 M) and 6 months (6 M). Weekly groupings are indicated at the top of the timeline (e.g., Week 1: S1–S3; Week 2: S4–S6). The intervention period is marked beneath the main timeline, with a follow-up period extending to the 3 M and 6 M timepoints. Each session involved balance testing under four conditions: Floor Eyes Open (EO), Floor Eyes Closed (EC), Foam EO, and Foam EC, each lasting 60 s. A zoomed-in schematic illustrates a typical session during the intervention period, where participants completed balance assessments before and after receiving either noisy electrical vestibular stimulation (nEVS) or Sham treatments. The 3-month and 6-month follow-ups included the same balance assessments without stimulation

The pilot study used a single-blind parallel randomized controlled trial design. Randomization was performed prior to recruitment using a computer-generated random sequence using atmospheric noise, created from a pre-specified list containing the group labels “Stimulation” (*n* = 24) and “Sham” (*n* = 24) in equal proportion. Participants in the Stimulation group received 20 min of continuous nEVS, while seated, whereas participants in the Sham group underwent identical protocol electrode placement and session procedures but no current was delivered beyond the initial brief low-amplitude discharge (“tap”) used to support blinding. The list was randomized to produce the final allocation order, which was then linked to sequential participation identification numbers. Participants were assigned the next available identification number in the order of enrollment. There was no allocation concealment mechanism beyond participant blinding. Additionally, two independent raters, who were not informed of group assignments, reviewed and removed movement artifacts from the standing balance data without participant identifiers (see Data Preparation section for more detail). On the final session, participants were asked to identify which group they believed they were in to assess potential bias. During the first visit, participants completed baseline questionnaires (see Questionnaire section for more detail). Participants attended three sessions per week for six weeks, with follow-ups at 3 months and 6 months post-treatment. The study timeline and procedures are summarized in Fig. 1. Each session lasted approximately 45 min. All procedures, including participation allocation, intervention administration, and outcome assessments, follow the pre-specified protocol. No study protocol or outcome changes were implemented after the trial commenced.

**Fig. 2:**
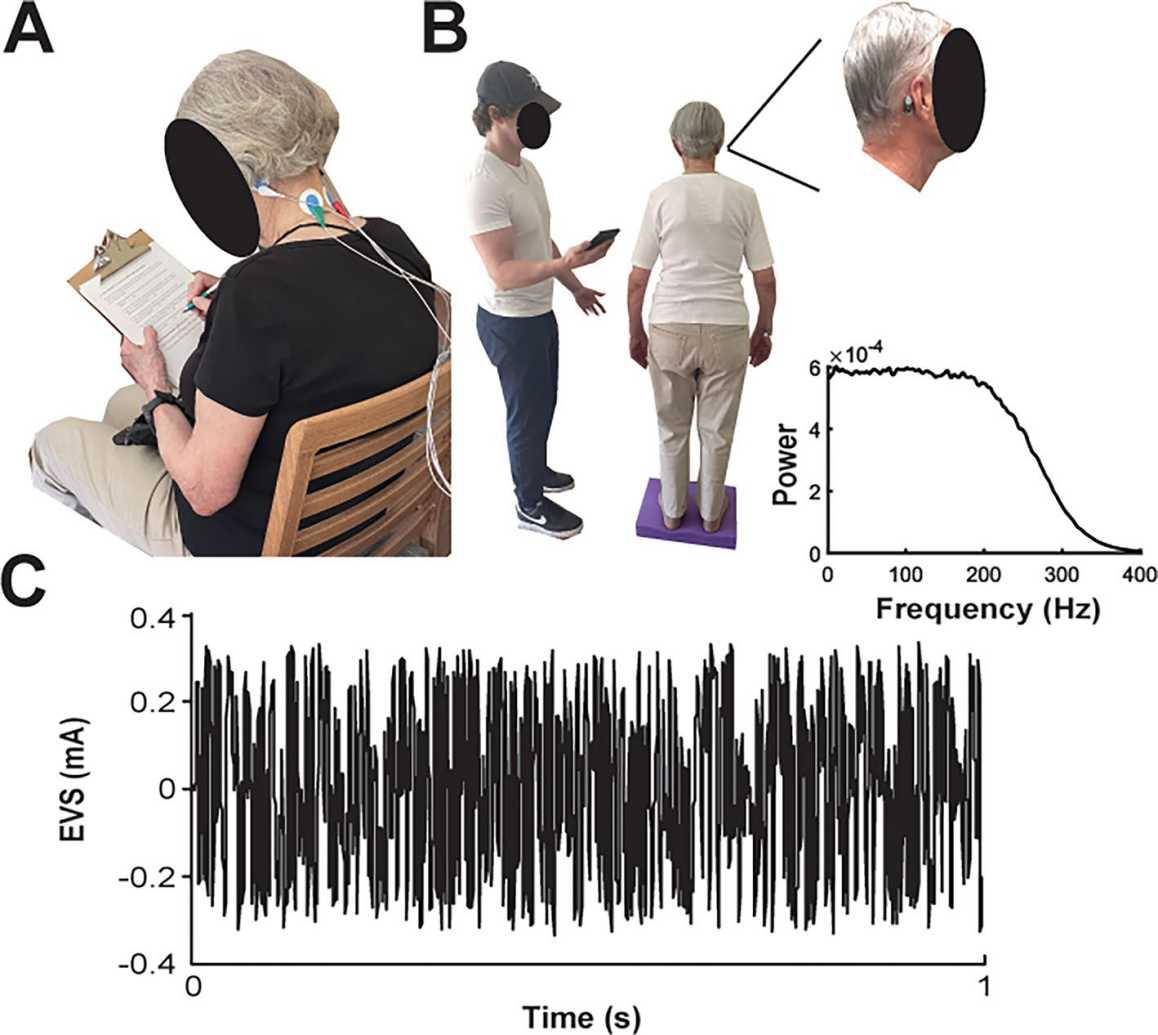
Experimental setup and data collection. **A** Participant sitting during noisy electrical vestibular stimulation (nEVS) while completing a questionnaire, with a 4-electrode bipolar configuration visible on the neck. **B** A participant performing a standing balance test on a soft surface, while the investigator measures standing balance using a Phybrata sensor located behind the right mastoid process, connected to a mobile app. **C** One second sample of nEVS waveform (± 0.35 mA, 0.001–300 Hz). The inset shows a power-spectral density plot for the 20-minute signal

Four balance assessments, described in more detail below, were administered via a smartphone app before and after stimulation. After the initial balance assessment, electrodes were placed bilaterally over the mastoid process and 2 cm from the midline at the C4 level on all participants (Fig. 2A). The Stimulation group received nEVS, while the Sham group did not. Participants were seated and not required to complete any specific or mental tasks during the 20 min of nEVS dosing. Immediately after nEVS, electrodes were removed, and participants repeated balance assessments. Follow-up sessions required participants to repeat the balance assessments twice, without the 20 min of nEVS or Sham dosing.

Participants removed footwear to complete the balance testing, which included a modified versions of the Romberg Test (a.k.a. modified Clinical Test of Sensory Interaction on Balance; mCTSIB), in which a head-mounted sensor was used to measure the participant’s ability to stand unassisted (Fig. 2B) under four test conditions designed to challenge the vestibular, visual, and proprioception sensory inputs at different levels [34, 35]. The four balance assessments, each 60 s in duration, were carried out in the following sequence: eyes open on a firm surface (Floor EO), eyes closed on a firm surface (Floor EC), eyes open on a soft surface (Foam EO), and eyes closed on a soft surface (Foam EC). The soft surface was a foam pad produced by Node Fitness (16 × 12 × 2.5 inches) used to reduce proprioceptive feedback from the feet and ankles [4]. For all tasks, participants were instructed to stand still with their chin parallel to the floor (neutral head posture), feet together, and arms at their sides. Investigators stood close to the participant during balance tasks for safety. To remove vision, participants closed their eyes, which was confirmed by the experimenter standing next to them.

### Electrical vestibular stimulation

The skin was cleaned with an alcohol swab before attaching the nEVS electrodes. Pre-gelled electrodes (3.8 by 4.2 cm; Ambu Blue Sensor SP, Ballerup, Denmark) were placed on both mastoid processes and on both sides of the neck beside the spine at the level of the C4 vertebrae bilaterally. Electrode impedance between each mastoid-C4 pair was typically between ~ 3–8 kOhms. A custom rechargeable battery-powered, dual-channel, isolated constant current stimulation device (Neursantys Inc, Calgary, AB) was used for delivering the nEVS. Participants in the Stimulation group received 20 min of continuous nEVS dosing at ± 0.35 mA (0.70 mA peak-to-peak amplitude) from 0.001 to 300 Hz. (Fig. 2C). The stimulation waveform was generated using custom LabVIEW software (National Instruments, 2021, Austin, TX). The current study utilized a uniform white noise signal which was band-pass filtered (4th order Butterworth) between 0.001 and 300 Hz, and output at a rate of 5 kHz from the stimulator. Previous research has indicated that individuals typically begin to perceive vestibular sensations around 0.9–1.0 mA, with cutaneous sensations such as tingling or prickling occurring at similar or slightly lower intensities (0.85 mA) [36]. Currents in the range of 0.2–0.5 mA, have been shown to produce measurable changes in balance control and activate cortical vestibular processing areas, indicating that sub-threshold stimulation can still effectively engage vestibular pathways [37, 38]. Based on this evidence, ± 0.35 mA was selected as an amplitude likely to produce a physiological effect while minimizing perceptibility to support the single-blinded study design. The majority of participants did not feel the stimulation based on self-report, however, there were some that could initially, and this sensation would wane over the first 1–2 min of stimulation. A stimulation bandwidth of 0.001–300 Hz was selected to engage the vestibular system while avoiding unnecessary frequencies beyond the physiological range of vestibular afferents. A functional MRI study has shown that nEVS delivered in a low-to-mid frequency range (0.1–250 Hz) produces greater activation of vestibular cortical regions compared to EVS at a fixed polarity [39]. Neurophysiological recordings in primates demonstrate that vestibular afferents can encode stimulus frequencies up to ~ 300 Hz with little benefit at higher rates [40]. One previous study has demonstrated significant postural stability improvements in older adults following a single session of nEVS at a current level of 0.4 mA and frequency range of 0.1–640 Hz [27]. Both the Stimulation and Sham groups in the present study underwent identical procedures, including electrode placement, session duration, and balance assessment. All participants also felt a low-amplitude current pulse (“tap”) delivered to the electrodes prior to starting the stimulation interval. The only difference was the stimulator was subsequently turned off for the Sham group after the delivery of the “tap” pulse.

### Accelerometry

Participants were instrumented with a wearable balance sensor (Phybrata sensor; PROTXX Inc.) placed vertically on their head above the right mastoid process. (Fig. 2B) [41]. The sensor contains an inertial measurement unit (IMU) that measures head acceleration in three orthogonal axes (anterior-posterior, x; superior-inferior, y; medial-lateral, z). Phybrata power is calculated as the combined magnitude of the accelerations along all three axes, providing a direction-independent measure of postural stability (Phybrata Power = Σ_t_ (X_*t*_^2^ + Y_*t*_^2^ + Z_*t*_^2^) * 1.3 * 0.01 * 9.81^2^) [42]. In the case where an anomalous motion artifact was removed (see Data Preparation below), this summed power value was normalized to a full 60s trial via multiplying by a factor of [60s / length of clipped trial]. This metric reflects the total power in the head motion and has been validated against gold-standard force plate and motion capture measures for assessing balance performance [42, 43]. Previous research has shown that Phybrata power is sensitive to changes in sensory conditions (eyes open vs. closed) and surface (firm vs. compliant), as well as balance impairments resulting from conditions resulting from concussions, spinal cord injuries, and aging [41–45]. Clinically, higher Phybrata power has been shown to quantify the progressive decline in postural control and increase in intrinsic fall risk in older adults [44]. The strong correlation with established indicators of fall risk supports the use of Phybrata power as a primary outcome measure.

**Fig. 3:**
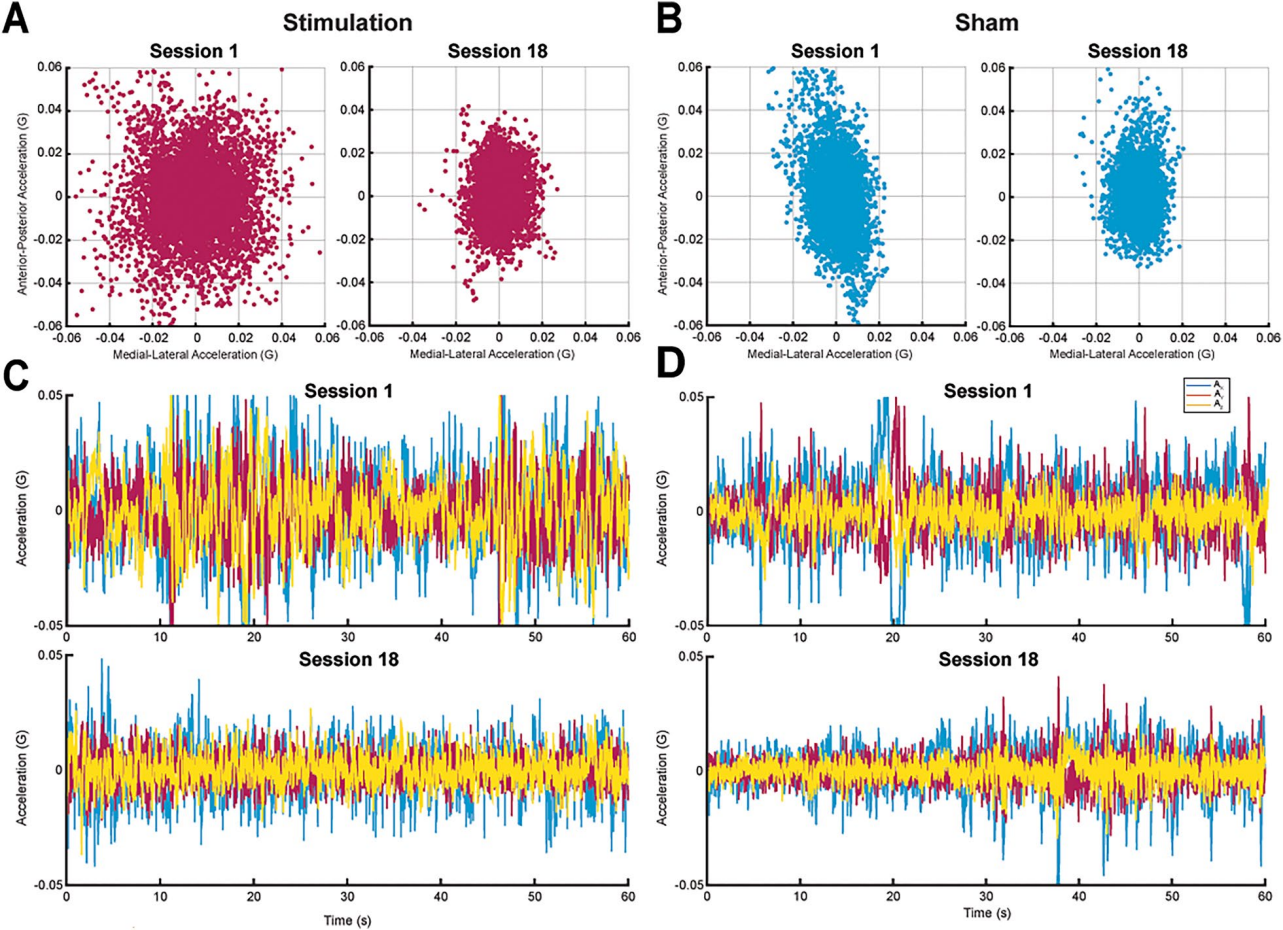
Example Phybrata data for the floor eyes closed condition on Session 1 and Session 18 for a Stimulation and a Sham participant. Phybrata data records over one minute sampled at 100 Hz. **A** Spatial scatter plot from a stimulation participant. The x-axis represents the medial-lateral acceleration (G), and y-axis represents the anterior-posterior acceleration (G) on Session 1 (left) and Session 18 (right). **B** Spatial scatter plots from a Sham participant. **C** Accelerometry timeseries for the stimulation participant, showing the acceleration in the x, y, and z directions on Session 1 (top) and Session 18 (bottom). **D** Acceleration timeseries for the Sham participant

The Phybrata sensor was attached to the skin with a disposable double-sided medical adhesive tape after cleaning the skin with an alcohol swab. Accelerometer data was recorded at 100 Hz and automatically relayed via a smartphone app to a cloud data system. Offline the raw accelerometer data was high pass filtered at 0.04 Hz (4th order Butterworth) to remove any DC offset. Phybrata sensor was removed after the balance assessments and during the stimulation interval. Figure 3 shows sample raw EO and raw EC raw acceleration time series data and spatial scatter plots collected before and after the nEVS treatment. Balance assessments were performed using the smartphone app developed by PROTXX Inc. The app guided the investigator and participant through the experimental procedures using on-screen instructions and auditory cues. All tests described in the Experimental Design section were completed using this app.

### Questionnaires

Five questionnaires were administered during the first visit: the Activity Balance Confidence Scale (ABC), the Symbol Digit Modality Test (SDMT), Headache Questions for Baseline, the Migraine Disability Assessment Scale (MIDAS), and the Physical Activity Scale for the Elderly (PASE). At the start of each session, participants were also asked if they had experienced any headaches since their previous visit and to rate their severity, with the results averaging over a month and compared between groups. Participants completed the ABC and MIDAS on the last session of the 6-week intervention. The ABC questionnaire measures an individual’s confidence in performing activities without losing balance. The ABC scale has been validated for older adults and is well-suited for identifying community-dwelling seniors beginning to lose confidence in their balance and who could benefit from a fall prevention intervention [46]. The SDMT is a general measure of attention, processing speed, visual scanning, and motor speed that requires test subjects to match numbers to symbols as quickly as possible for 90 s [47]. The validity and reliability of this test has been assessed for many different populations including older adults [47]. The PASE is a reliable and validated research measure of physical activities commonly engaged in by older adults [48]. Headache monitoring was included as an exploratory outcome based on interest in the potential for nEVS to affect headache or migraine symptoms. The MIDAS is a five-item questionnaire designed to measure headache-related disability and it demonstrates good test-retest reliability across population-based samples in the United States and United Kingdom [49].

### Data preparation

To ensure the accuracy and reliability of the data, two independent raters blind to subject groups manually reviewed the raw acceleration data for all testing sessions. Using a custom MATLAB script (MathWorks, R2024a, Natick, MA), any movement artifacts in the head acceleration data attributed to anomalous movements (e.g. stepping, talking, head-turning, shrugging, arm movements, hand fidgeting, coughing, sneezing, dozing off, shift in gaze with minor head movement), were identified and clipped from the data. Artifacts were clipped manually such that they started and ended at zero-acceleration crossings, so that when segments were concatenated afterwards there was no discontinuity in the acceleration profiles. This process ensured that the data used in the analysis represented actual standing balance performance, free from artifacts. A total of 1.94% of the entire dataset was clipped due to the occurrence of artifacts in the recordings. Outliers were identified and removed from the dataset to avoid their influence on the analysis. Using the Explore option in SPSS, outlier sessions were flagged based on extreme values in the positive or negative direction for each session, condition, and group. SPSS identifies outliers by calculating values that fall beyond 1.5 times the interquartile range from the first or third quartile. Additionally, cases were excluded if sensor or data uploading errors occurred during data collection (39 out of 7467, 0.52%).

### Statistical tests

All statistical tests were analyzed using IBM SPSS (v28.0.1.0, IBM Corp., 2021, Armonk, NY) with an alpha level of 0.05 for the statistical significance threshold of all testing. A formal a priori sample size calculation was not performed prior to data collection. Given there is no previous study of this sort to use as the basis for a sample size calculation, we instead determined our sample size based on feasibility constraints, including testing capacity and available equipment and human resources. For each session and conditions (floor and foam/ EO and EC), the Phybrata power used in the analyses were calculated as the average of the corresponding pre-nEVS and post-nEVS values. Data was normalized across the study to each individual’s initial session performance, which expresses relative change from baseline and accounts for large inter-subject differences in baseline balance performance. To determine the therapeutic efficacy of repeated nEVS sessions, Phybrata power was analyzed using a linear mixed model analysis with subjects as the random factor, and group (Stimulation or Sham), session (Session 1 to 18), and interaction of group and session as fixed factors. Planned pairwise comparisons were used to compare Session 1’s performance to the subsequent sessions. A linear mixed model is best suited for this data analysis because it can compare data across multiple time points and handle missing data. Regressions comparing Week 1 scores (average of 6 tests, pre/post for Sessions 1, 2, 3) and change in performance (Week 6 scores – Week 1 scores) were conducted on all conditions to evaluate whether baseline balance ability predicted the magnitude of improvement over the intervention period. Pearson’s Correlation (two-tailed) analyses were conducted to examine the relationship between Week 1 scores, Week 6 scores (average for Sessions 16, 17, 18), and change in performance (Week 6 scores – Week 1 scores) across all conditions, with the Stimulation and Sham group analyzed separately, against the questionnaire data (Week 1 scores with PRE ABC, Week 6 scores with POST ABC, change in performance vs. change in ABC score (POST ABC – PRE ABC), and Week 1/Week 6/change in performance vs. SDMT and PASE). T-tests were used to assess changes and differences between groups. Paired t-tests compared PRE ABC and POST ABC for both groups. Independent t-tests were performed to compare headache frequency between both groups. The effectiveness of the single-blind study design was tested with crosstabulation using a chi-squared test to determine whether the distribution of correct and incorrect guesses regarding participants assigned group (Stimulation or Sham) differed from expected. Independent t-tests and chi-squared test were used to compare Stimulation and Sham group baseline characteristics.

## Results

**Fig. 4:**
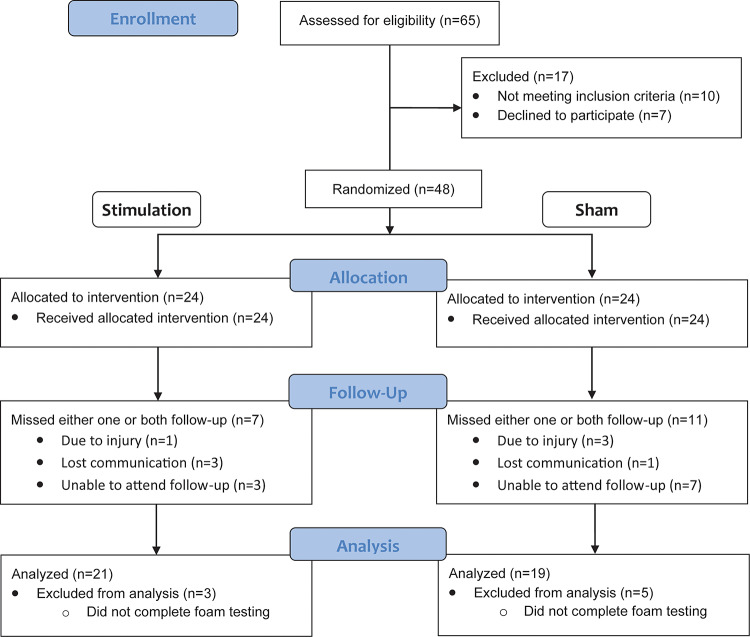
CONSORT flow diagram for participation recruitment, allocation, follow-up, and analysis. A total of 65 participants were assessed for eligibility, with 10 excluded due to failure to meet the inclusion criteria and 7 excluded due to declining to participate. The remaining 48 participants were randomized into the Stimulation group (*n* = 24) and Sham group (*n* = 24). All participants received their allocated intervention. However, 7 participants were lost to follow-up in the Stimulation group, and 11 participants were lost to follow-up in the Sham group. After applying exclusion (completing all testing conditions), 21 participants were analyzed in the Stimulation group and 19 participants in the Sham group

Participants were recruited between October 2022 and November 2023 from retirement homes, assisted living facilities and community residents around the University of Calgary. Out of the 48 participants initially enrolled in the study, only those who completed all testing sessions were included in the final analysis. This criterion resulted in a total of 40 participants (14 males and 26 females) (19 Sham, 21 Stimulation) whose data were analyzed. Participants who were unable to complete the foam testing were excluded, which accounted for the removal of 8 participants (5 Sham, 3 Stimulation). This approach ensured consistency across participants for all conditions and testing sessions. See Fig. 4 for CONSORT flow diagram of how participants flowed through the study. The final sample of 40 older adults had a mean age of 77.7 ± 11.8 years, height: 167.8 ± 10.8 cm, and weight: 70.7 ± 14.0 kg. Table 1 displays baseline demographics, questionnaire, and balance of Stimulation and Sham groups. The study was completed as originally intended, with all planned assessments and follow-ups carried out as per the protocol. No adverse effects of the intervention were reported.

**Table 1:** Baseline demographic, clinical, and balance performance characteristics by groups

Variable	Stimulation (*n* = 21)	Sham (*n* = 19)	*p*-value	Test
*Demographics*				
Age (years)	74.4 ± 12.5	78.3 ± 12.2	0.33	t-test
Height (cm)	168.5 ± 13.1	167.0 ± 9.2	0.68	t-test
Weight (kg)	70.2 ± 14.7	68.7 ± 14.7	0.72	t-test
Sex (M / F)	7 / 14	7 / 12	0.82	χ² test
Testing location (lab / residential)	12 / 9	7 / 12	0.20	χ² test
*Baseline Scores*				
PASE Score	142.6 ± 83.4	109.9 ± 93.1	0.26	t-test
ABC Score (%)	79.6 ± 19.7	79.8 ± 19.2	0.98	t-test
SDMT (symbols correct)	36.8 ± 12.6	34.9 ± 12.4	0.66	t-test
*Baseline Phybrata Power (Raw)*				
Floor EO (Session 1)	0.46 ± 0.44	0.42 ± 0.22	0.71	t-test
Floor EC (Session 1)	0.64 ± 0.55	0.53 ± 0.41	0.46	t-test
Foam EO (Session 1)	2.30 ± 2.60	1.48 ± 1.02	0.20	t-test
Foam EC (Session 1)	6.74 ± 5.32	6.66 ± 8.37	0.97	t-test

Values are presented as mean ± standard deviation unless otherwise noted. This table summarizes baseline demographic (age, height, weight, sex, testing location), clinical (PASE, ABC, SDMT), and balance performance measures for the Stimulation and Sham groups. Group differences were analyzed using independent samples t-tests for continuous variables and chi-square (χ²) tests for categorical variables. Phybrata power is shown for each of the four balance conditions during Session 1: Floor Eyes Open (EO), Floor Eyes Closed (EC), Foam EO, and Foam EC. No significant between-group differences were found at baseline across demographic, clinical, or balance performance variables (all *p* > 0.05)

*ABC* Activities-specific Balance Confidence; *SDMT* = Symbol Digit Modalities Test; *PASE* = Physical Activity Scale for the Elderly; *EO* = Eyes Open; *EC* = Eyes Closed

### Retention

Participant retention diminished after the 6-week intervention. Of the 40 participants 27 completed the 3-month follow-up and 30 completed the 6-month follow-up. If a participant was unable to attend a scheduled follow-up, they were still permitted to complete later follow-ups as their schedule allowed.

### Blinding

Of the 15 participants assigned to the Sham group and retained to the 6-month follow-up session, 53.3% (*n* = 8) correctly identified their group, while 46.7% (*n* = 7) incorrectly thought they were in the Stimulation group. Conversely, of the 18 participants in the Stimulation group, 72.2% (*n* = 13) correctly identified their group, while 27.8% (*n* = 5) thought they were in the Sham group. A chi-square test was performed to assess whether participants’ perceptions of their group assignment were associated with their actual group assignment. The test was not statistically significant, χ²(1, *N* = 33) = 2.238, *p* = 0.135, indicating no evidence of an association between group assignment and perceived group.

### nEVS reduces phybrata power in older adults compared to controls

**Fig. 5:**
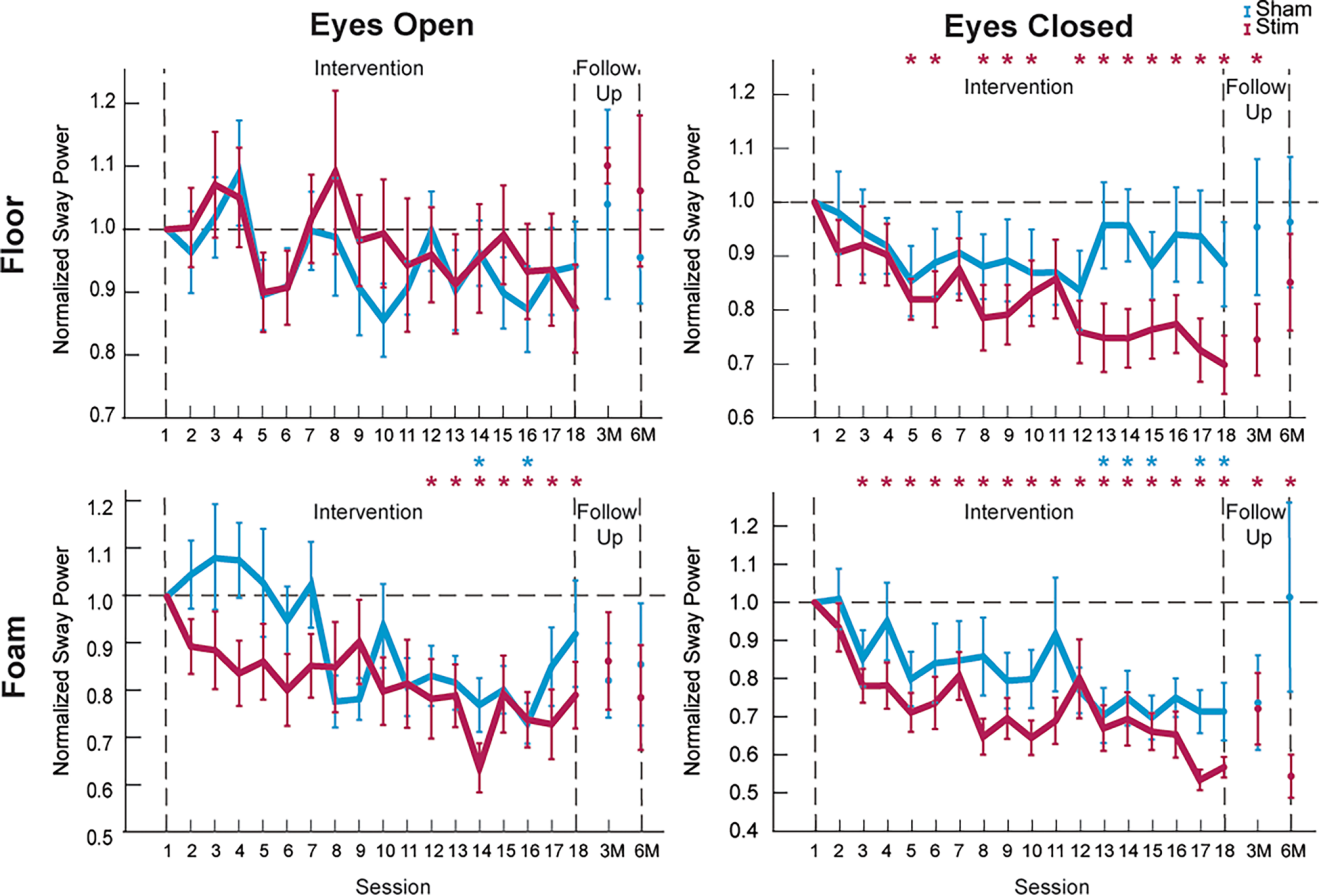
Group-level comparisons of normalized Phybrata power across the nEVS intervention and follow-up periods under four conditions: **A** Floor Eyes Open (EO), **B** Floor Eyes Closed (EC), **C** Foam EO, and **D** Foam EC. Balance performance is separated by group, with the Sham group as blue and the Stimulation group as red. The x-axis denotes the sessions of the intervention (Session 1–18), followed by mean data points for 3-Month and 6-Month follow-up. Error bars represent ± 1 standard error. A dashed line at y = 1 indicates normalized performance relative to Session 1, where data represent Phybrata power ratios (e.g., Session 2 Phybrata power = Session 2/Session 1). Note that the y-axis scale differs between panels to appropriately display the data

See Additional file 1 (Table) for mean +/- SD for each group in the linear mixed model (LMM). In the LMM analysis, significant main effects of group were observed in Floor EC (F(1,635) = 19.427, *p* < 0.001), Foam EO (F(1,599) = 23.701, *p* < 0.001), and Foam EC (F(1,610) = 17.560, *p* < 0.001) conditions, indicating differences between the Stimulation and Sham groups across conditions. Significant main effect of session was observed in the Foam EO (F(17,599) = 3.148, *p* < 0.001) and Foam EC (F(17,610) = 3.947, *p* < 0.001) conditions suggesting changes in performance over time. No significant interaction effects (group * session) were observed for any condition, including Floor EO (F(17,616) = 0.244, *p* = 0.999), Floor EC (F(17,635) = 0.692, *p* = 0.813), Foam EO (F(17,599) = 0.969, *p* = 0.492), Foam EC (F(17,610) = 0.537, *p* = 0.935). These results indicate that the Stimulation group improved more than the Sham group, however, both groups demonstrated improvements across testing sessions. Figure 5 shows the times series for Floor EO, Floor EC, Foam EO, and Foam EC for the Stimulation and Sham groups.

### Pairwise comparisons

Table 2 shows p-values of pairwise comparisons of subsequent sessions to Session 1 with an asterisk denoting significance. Pairwise comparisons for the Floor EO condition of all sessions compared to Session 1 show no significant differences for both the Stimulation and Sham groups (all *p* > 0.05). In the Floor EC condition, the Sham group exhibited no significant differences in performance across all pairwise comparisons with Session 1 (all *p* > 0.05), suggesting no meaningful change over time. Conversely, the Stimulation group demonstrated significant reductions in normalized Phybrata power starting on Session 5 compared to Session 1 (Mean Diff. = − 0.180, *p* = 0.03), with progressively larger reductions observed through Session 18 (Mean Diff = − 0.302, *p* < 0.001), except Sessions 7 and 11 (*p* > 0.05). By the 6-month follow up, the reduction in normalized Phybrata power diminished slightly (Mean Diff = − 0.149, *p* = 0.092). In the Foam EO condition, the Sham group exhibited minimal changes in performance relative to Session 1, with significant improvements in normalized Phybrata power observed on Session 14 (Mean Diff = − 0.229, *p* = 0.036), and Session 16 (Mean Diff = − 0.268, *p* = 0.018). In contrast, the Stimulation group displayed earlier and more consistent improvements in normalized Phybrata power, with significant reductions first observed on Session 12 (Mean Diff = − 0.216, *p* = 0.034). These significant improvements persisted through Session 18 (Mean Diff = − 0.208, *p* = 0.043) but were not significant at the 3- and 6-month follow ups (*p* > 0.05). In the Foam EC condition, the Sham group exhibited significant improvements compared to Session 1 performance starting on Session 13 and persisting through Session 18 except for Session 16 and 3- and 6-month follow ups (*p* > 0.05). The Stimulation group demonstrated earlier and more sustained improvements in normalized Phybrata power, with significant differences starting at Session 3 (Mean Diff = − 0.219, *p* = 0.006) and persisting through the 6-month follow-up (Mean Diff = − 0.456, *p* < 0.001).

**Table 2:** P-values for pairwise comparisons of phybrata power vs. Session 1 across groups and conditions

Session (vs. Session 1)	Floor EO		Floor EC		Foam EO		Foam EC	
	Sham	Stim	Sham	Stim	Sham	Stim	Sham	Stim
2	0.673	0.979	0.844	0.273	0.658	0.317	0.946	0.403
3	0.835	0.516	0.589	0.348	0.436	0.277	0.243	0.006*
4	0.302	0.646	0.445	0.240	0.464	0.131	0.694	0.005*
5	0.244	0.370	0.162	0.030*	0.784	0.193	0.115	< 0.001*
6	0.303	0.394	0.275	0.028*	0.651	0.071	0.205	< 0.001*
7	0.978	0.878	0.354	0.128	0.814	0.160	0.220	0.012*
8	0.889	0.411	0.245	0.012*	0.056	0.153	0.259	< 0.001*
9	0.291	0.869	0.287	0.011*	0.057	0.351	0.108	< 0.001*
10	0.123	0.952	0.210	0.039*	0.568	0.055	0.122	< 0.001*
11	0.297	0.615	0.206	0.086	0.086	0.078	0.496	< 0.001*
12	0.974	0.710	0.107	0.004*	0.133	0.034*	0.067	0.010*
13	0.281	0.425	0.677	0.002*	0.091	0.040*	0.017*	< 0.001*
14	0.661	0.673	0.675	0.002*	0.036*	< 0.001*	0.046*	< 0.001*
15	0.251	0.936	0.252	0.004*	0.072	0.048*	0.017*	< 0.001*
16	0.149	0.538	0.558	0.007*	0.018*	0.016*	0.051	< 0.001*
17	0.454	0.555	0.537	< 0.001*	0.176	0.01*	0.025*	< 0.001*
18	0.523	0.253	0.270	< 0.001*	0.471	0.043*	0.023*	< 0.001*
3 M	0.704	0.428	0.711	0.007*	0.146	0.230	0.062	0.001*
6 M	0.678	0.608	0.759	0.092	0.240	0.051	0.923	< 0.001*

This table presents p-values from pairwise comparisons between each session (Sessions 2–18, 3-month [3 M], 6-month [6 M]) and Session 1 for Phybrata power across four balance conditions: Floor Eyes Open (EO), Floor Eyes Closed (EC), Foam EO, Foam EC. Results are shown separately for the Stimulation and Sham groups. Asterisks (*) and bold denote significant differences relative to Session 1 with α = 0.05 level

### Regression analysis

**Fig. 6:**
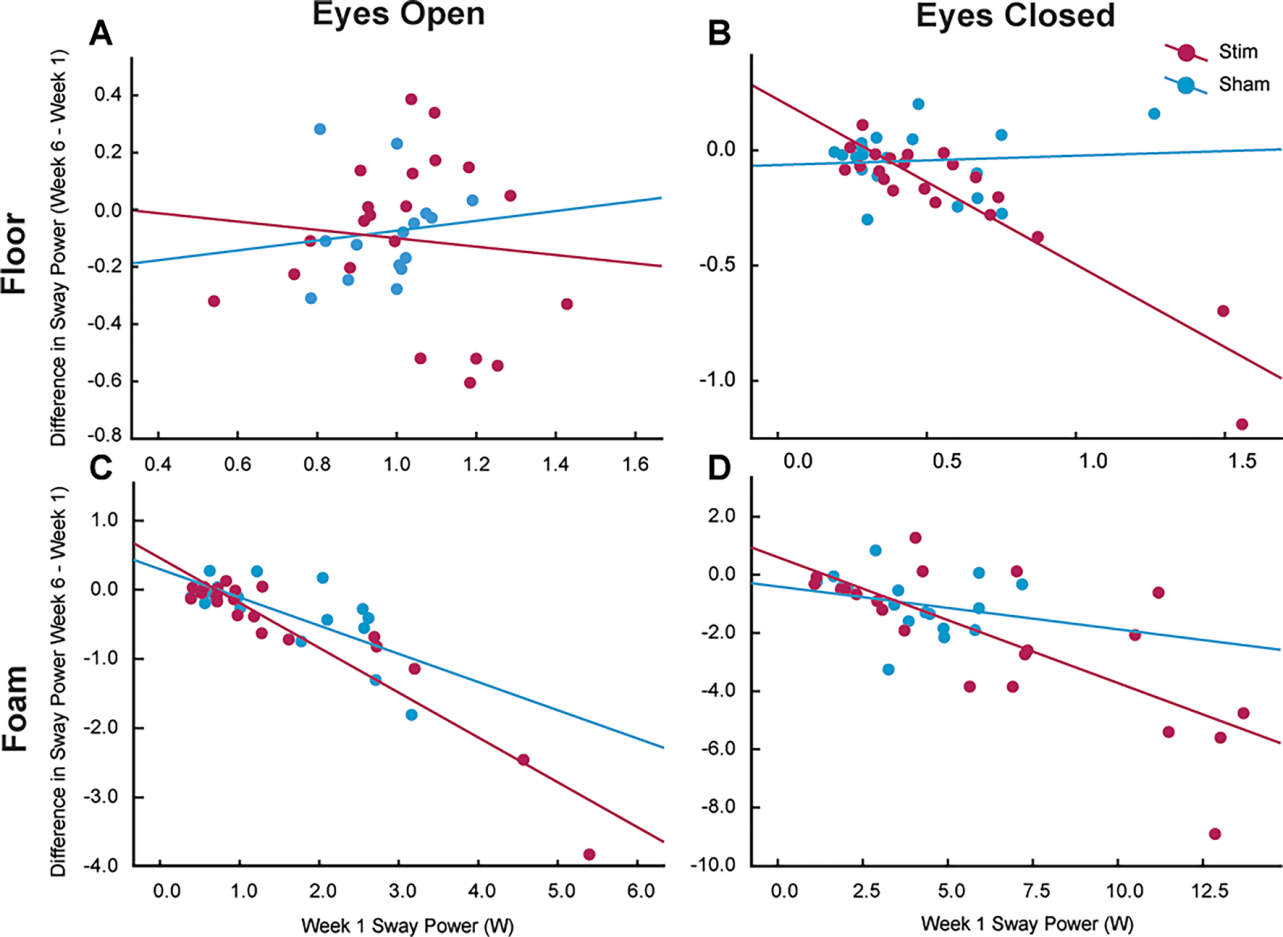
Correlation between Week 1 performance and change in Phybrata power over the intervention period (Week 6 – Week 1) for four conditions: **A** Floor Eyes Open (EO), **B** Floor Eyes Closed (EC), **C** Foam EO, and **D** Foam EC. Negative values in the change in Phybrata power indicate improvements in balance performance throughout the intervention. Blue represents the Sham group, and red represents the Stimulation group, with lines of best fit shown for each group

In the Floor EO conditions, neither group demonstrated a significant relationship between baseline performance and change over the intervention period (Sham: F(1, 14) = 0.19, *p* = 0.670, R² = 0.013; Stimulation: F(1, 19) = 0.20, *p* = 0.659, R² = 0.010), suggesting limited predictive value of initial performance in this low-challenged condition. For Floor EC, baseline performance significantly predicted change in performance for the Stimulation group (F (1, 19) = 97.40, *p* < 0.001, R² = 0.837). Baseline scores negatively predicted improvement (B = − 0.716, *p* < 0.001), indicating that participants with poorer initial balance improved the most. The Sham group did not demonstrate a significant relationship (F (1, 17) = 0.105, *p* = 0.750, R² = 0.006). In Foam EO, significant relationships were observed for both Stimulation groups (F(1, 18) = 142.02, *p* < 0.001, R² = 0.888) and Sham (F(1, 16) = 16.66, *p* < 0.001, R² = 0.510), with larger baseline balance impairments predicting larger balance improvements (B = − 0.648, *p* < 0.001 for Stimulation; B = − 0.407, *p* < 0.001 for Sham). Lastly, in the Foam EC condition, baseline performance was a significant predictor of change in performance for only the Stimulation group (F(1,19) = 22.87, *p* < 0.001, R^2^ = 0.546) with larger Session 1 balance impairments again predicting greater balance improvements (B = − 0.432, *p* < 0.001), while the Sham group did not exhibit a significant relationship (F(1,15 = 1.03, *p* = 0.326, R² = 0.254, B = − 0.147). Figure 6 shows scatterplots of the Floor EO, Floor EC, Foam EO, and Foam EC Week 1 performance vs. change in performance (Week 6 – Week 1).

**Table 3:** Correlations between balance outcomes and questionnaire scores across conditions and timepoints

Group	Condition	Measure Type	Questionnaire	*r*	*p*
Sham	Floor EO	Baseline (W1)	PreABC	0.488	**0.047**
			PASE	0.034	0.896
			SDMT	0.365	0.149
		Post (W6)	PostABC	0.266	0.303
			PASE	0.008	0.977
			SDMT	− 0.059	0.828
		Change in performance	ChangeinABC	0.16	0.541
			PASE	− 0.016	0.953
			SDMT	− 0.53	0.844
	Floor EC	Baseline (W1)	PreABC	− 0.548	0.49
			PASE	− 0.087	0.731
			SDMT	− 0.072	0.776
		Post (W6)	PostABC	− 0.47	**0.049**
			PASE	− 0.105	0.679
			SDMT	− 0.012	0.965
		Change in performance	ChangeinABC	0.02	0.936
			PASE	− 0.062	0.806
			SDMT	0.159	0.529
	Foam EO	Baseline (W1)	PreABC	− 0.65	**0.003**
			PASE	− 0.287	0.249
			SDMT	− 0.377	0.123
		Post (W6)	PostABC	− 0.588	**0.01**
			PASE	− 0.244	0.329
			SDMT	− 0.356	0.16
		Change in performance	ChangeinABC	0.169	0.502
			PASE	0.196	0.435
			SDMT	0.24	0.354
	Foam EC	Baseline (W1)	PreABC	− 0.617	**0.011**
			PASE	− 0.341	0.197
			SDMT	0.144	0.595
		Post (W6)	PostABC	− 0.329	0.182
			PASE	− 0.378	0.122
			SDMT	0.23	0.375
		Change in performance	ChangeinABC	0.211	0.434
			PASE	0.106	0.696
			SDMT	− 0.057	0.834
Stim	Floor EO	Baseline (W1)	PreABC	− 0.098	0.681
			PASE	0.08	0.73
			SDMT	0.178	0.454
		Post (W6)	PostABC	− 0.209	**− 0.39**
			PASE	− 0.022	0.925
			SDMT	0.456	**0.043**
		Change in performance	ChangeinABC	0.255	0.292
			PASE	− 0.081	0.726
			SDMT	0.406	0.075
	Floor EC	Baseline (W1)	PreABC	− 0.31	0.184
			PASE	− 0.159	0.492
			SDMT	− 0.235	0.318
		Post (W6)	PostABC	− 0.063	0.798
			PASE	− 0.34	0.132
			SDMT	− 0.122	0.62
		Change in performance	ChangeinABC	0.296	0.218
			PASE	0.018	0.937
			SDMT	0.234	0.322
	Foam EO	Baseline (W1)	PreABC	0.365	0.114
			PASE	− 0.178	0.441
			SDMT	− 0.405	0.077
		Post (W6)	PostABC	− 0.228	0.363
			PASE	− 0.224	0.342
			SDMT	− 0.349	0.143
		Change in performance	ChangeinABC	0.05	0.843
			PASE	0.026	0.915
			SDMT	0.292	0.224
	Foam EC	Baseline (W1)	PreABC	− 0.247	0.294
			PASE	− 0.283	0.214
			SDMT	− 0.142	0.551
		Post (W6)	PostABC	− 0.092	0.709
			PASE	− 0.256	0.264
			SDMT	− 0.222	0.347
		Change in performance	ChangeinABC	0.121	0.623
			PASE	0.182	0.43
			SDMT	− 0.019	0.935

This table summarizes the Pearson correlation coefficients (r) and associated p-values between balance performance and questionnaire scores, stratified by group (Stimulation vs. Sham), balance condition (Floor Eyes Open (EO), Floor Eyes Closed (EC), Foam EO, Foam EC), and timepoint (Week 1 Baseline, Week 6 Post, or Change in Performance [week 6 – week 1 scores]). Balance was quantified using Phybrata power, and questionnaire measures included the Activities-specific Balance Confidence Scale (ABC; Pre-, Post-, and change in score), the Physical Activity Scale for the Elderly (PASE), and the Symbol Digit Modalities Test (SDMT)

### Questionnaire analysis

An independent t-test comparing headache frequency between the Stimulation and Sham groups found no significant difference (*p* = 0.473) throughout the 6-week intervention. There were no significant differences in MIDAS scores from pre- to post-intervention for the Stimulation group (*p* = 0.302) and the Sham group (*p* = 0.363). There was a significant increase in balance confidence scores from pre- to post-intervention in both the Stimulation (*p* = 0.023) and Sham groups (*p* = 0.019). Pearson correlations between balance performance and questionnaire scores (ABC, PASE, SDMT) are summarized in Table 3. Across both groups and all four conditions, the majority of relationships were not statistically significant. In the Sham group, baseline performance in Floor EO was positively correlated with pre-intervention ABC scores (*r* = 0.488, *p* = 0.047), and baseline performance in Foam EO and Foam EC was negatively correlated with pre-intervention balance confidence (*r* = − 0.650, *p* = 0.003; *r* = − 0.617, *p* = 0.011, respectively). The stimulation group showed a significant correlation between post-intervention Floor EO balance and SDMT scores (*r* = 0.456, *p* = 0.043) No consistent relationships emerged across other conditions or groups. A strong negative correlation was identified between age and SDMT scores (*r* = − 0.799, *p* < 0.001), demonstrating that older participants exhibited lower cognitive performance. Age was also negatively correlated with balance confidence (*r* = − 0.545, *p* < 0.001), suggesting that older participants reported lower confidence in performing balance-related tasks. There were no significant relationships between SDMT scores and balance performance.

## Discussion

This study demonstrates that cumulative nEVS dosing is a novel and potentially effective therapeutic approach for improving balance in older adults. Relative to the Sham group, the Stimulation group showed earlier, larger, and more persistent reductions in Phybrata power. In Floor EC, Stimulation group improvement emerged by Session 5 (but not Sessions 7 and 11), with benefits evident at 3-month (but not 6-month) follow up. In Foam EO, Stimulation improvements began at Session 12 and persisted through Session 18 but were not maintained at 3 or 6 months. In Foam EC, Stimulation improvement began by Session 3 and persisted through both the 3- and 6-month follow-ups. In contrast, the Sham group demonstrated either delayed or inconsistent improvements, with occasional significant changes appearing only in the latter half of the intervention (e.g., Session 14 and 16 in Foam EO), consistent with learning or practice effects from repeated balance assessments. These trends suggest that the improvement observed in the Stimulation group are unlikely due to placebo or task familiarity alone but rather reflect the additive therapeutic effects of repeated nEVS. The absence of change in the simpler Floor EO task for both groups suggest nEVS benefits are most apparent when balance relies more heavily on vestibular and proprioceptive integration. Together, these findings support nEVS as a minimally invasive, portable intervention targeting age-related balance deficits.

We did not include a formal VRT arm in this pilot trial because our primary objective was to evaluate the feasibility and mechanistic effects of cumulative nEVS. Specifically, we aimed to determine whether repeated nEVS alone could induce measurable improvements in balance and to identify when and under which sensory conditions these improvements emerge. Including a VRT arm will be an important next step in future trials to evaluate the additive or synergistic potential of nEVS when combined with established therapeutic approaches.

### Potential central mechanisms

The potential central mechanisms underlying the improvements in balance observed in this study may involve neuroplasticity within the vestibular nuclei and associated neural pathways. The vestibular nuclei are critical for processing sensory inputs from the vestibular system and integrating these signals with proprioceptive and visual information to maintain postural control. Neuroplasticity within these nuclei may play a key role in the sustained balance improvements observed in participants receiving nEVS.

Evidence from previous studies supports the potential for long-term potentiation (LTP) within the vestibular nuclei, a neural mechanism that enhances synaptic efficacy [50]. This finding aligns with the hypothesis that repeated nEVS sessions may promote LTP in the vestibular nuclei, thereby improving the processing of vestibular inputs. The vestibular nuclei has capacity for neuroplasticity, particularly following vestibular stimulation or injury [51, 52]. Neuroplasticity localized to the synapse between afferents and the vestibular nuclei has been demonstrated in animal research showing an altered patterning of synaptic potentials and expression of NMDA and AMPA receptors at this synapse post-electrical stimulation [53]. These studies suggest that repeated nEVS exposure may facilitate functional changes in the vestibular nuclei, enhancing their ability to process and respond to sensory inputs.

Vestibular neuroplasticity, specifically synaptic plasticity within the vestibular nuclei, may account for the prolonged balance improvements observed following nEVS [26]. Significant post-stimulation effects lasting several hours have been observed in healthy older adults, supporting the idea that nEVS induces neuroplastic changes in the central vestibular pathways [26]. This aligns with our study’s findings, where participants in the Stimulation group exhibited sustained improvements in balance control, with effects persisting through the 6-month follow-up. The extended duration of these improvements suggests that cumulative nEVS may induce long-lasting neuroplastic changes in the central vestibular system. Multiple studies have now shown that subliminal passive body motion (linear translation and yaw rotation) can induce improved vestibular sensitivity [54–56]. It is argued that the results of these studies demonstrate bi-directional homeostatic plasticity, a regulatory response of the central nervous system to low-intensity stimulation that can increase neural responsiveness.

Additionally, the cerebellum, which is closely linked to the vestibular nuclei, likely contributes to the observed effects. The cerebellum plays a crucial role in fine-tuning motor control and balance by integrating sensory inputs and coordinating motor responses. Neuroplasticity within the cerebellum, particularly in its connections with the vestibular nuclei, may enhance the central processing of vestibular inputs. nEVS can activate cortical areas beyond the vestibular nuclei, including regions associated with motor planning and balance control [38]. While our study employed subthreshold nEVS levels that were unlikely to evoke direct cortical activation, the observed improvements suggest that repeated dosing may strengthen vestibular pathways.

Age-related reductions in vestibular nuclei neurons and cerebellar function may further highlight the importance of nEVS-induced neuroplasticity in older adults. Aging leads to neuronal loss in the vestibular nuclei, contributing to declines in balance performance [10, 57]. nEVS may counteract these effects by enhancing synaptic efficacy and promoting neuroplastic changes within central pathways, allowing older adults to maintain or improve postural stability. In addition to enhancing balance, nEVS may also have broader implications for central neural function. Higher EVS input levels activate cortical areas involved in visual and motor integration, suggesting that repeated dosing could improve sensory integration and motor planning [38]. While our study focused on subthreshold stimulation, future research should investigate whether higher nEVS intensities could amplify these central effects.

### Potential peripheral mechanisms

However, we cannot rule out that repeated exposure to nEVS in this study caused peripheral adaptations at the level of vestibular hair cells and their afferents. Vestibular afferents, which transmit signals from the vestibular sensory organs to the brain, are crucial for maintaining postural stability. Prior research supports the idea that EVS directly influences vestibular afferent activity, enhancing the processing of vestibular signals necessary for balance control [23, 40]. For instance, Goldberg et al. (1984) demonstrated that intra-vestibule EVS modulates vestibular afferent firing, specifically from the otolith organs and semicircular canals [58]. This finding suggests that EVS enhances the input generation of peripheral vestibular signals to the vestibular nuclei, thereby improving postural stability during challenging balance tasks [58].

The findings from Kwan et al. (2019) further reinforce this notion by showing that transcranial EVS activates both semicircular canal and otolith afferents in nonhuman primates [22]. This balanced activation of vestibular afferents may account for the improvements in sensory integration observed in participants performing tasks that rely heavily on vestibular and proprioceptive inputs, such as the Floor EC and Foam EC conditions. By increasing the sensitivity of vestibular afferents to subthreshold signals, nEVS likely enhances the neural inputs that enable detection of subtle head and body movements to maintain postural stability. Additionally, it is proposed that nEVS-induced improvements in postural stability may be driven by stochastic resonance, a phenomenon in which noise added to a system enhances signal detection [59]. However, stochastic resonance should only occur during stimulation, and cannot account for the persistent effects observed here with repeated nEVS exposure. The cumulative effects of nEVS dosing may amplify vestibular afferent inputs by strengthening synaptic gain to between the hair cells and their afferents, as well as between the afferents and vestibular nuclei leading to pronounced improvements in vestibular balance control [53]. Detecting and quantifying the contributions of such neuroplastic changes at the synapse between the hair cell and vestibular afferents with electrical stimulation remains an important avenue for future research. Participants in the Stimulation group showed significant reductions in Phybrata power as early as Session 3 in the Foam EC condition, showing the rapid impact of nEVS on peripheral vestibular processing.

Age-related changes in vestibular hair cell density and function may further explain the effectiveness of nEVS in older adults. nEVS not only stimulates vestibular afferents but may also directly influence the function of vestibular hair cells [60]. Although the capacity for vestibular hair cell regeneration in humans is limited, immature hair bundles have been observed in the utricular epithelia of adults over 60 years old, suggesting a retained capacity for regeneration [12]. This regeneration may occur through direct transdifferentiation of supporting cells into hair cells or via asymmetric division, where one daughter cell becomes a hair cell and the other remains a supporting cell [61]. nEVS may stimulate these immature hair bundles, potentially supporting functional recovery of vestibular sensory cells. Further research is required to validate whether nEVS can directly facilitate hair cell regeneration or if its effects are limited to afferent stimulation. The modulation of afferent firing by nEVS may also address the age-related decline in vestibular sensitivity. Vestibular hair cell loss contributes to reduced sensory input in older adults, leading to balance impairments [10]. By enhancing vestibular afferent sensitivity and potentially stimulating remaining hair cells, nEVS could compensate for this sensory loss, allowing older adults to maintain or improve postural stability under challenging conditions.

### Clinical significance

This study demonstrates that nEVS may be a promising therapeutic tool for improving balance in older adults, particularly in conditions that rely heavily on vestibular and proprioceptive inputs. Improvements observed in the Stimulation group, especially in the Foam EC and Foam EO conditions, emphasize its utility in targeting sensory integration processes critical for postural stability. Although participants with clinically diagnosed vestibular disorders were excluded to isolate the effects of nEVS to normal aging populations, it is well established that peripheral and central vestibular function diminishes with age, and that vestibular dysfunction may present long before symptoms emerge or are diagnosed [8, 11]. As such, our sample likely included individuals with subclinical or undiagnosed vestibular hypofunction. Peripheral vestibular deficits are prevalent among older populations and exacerbate age-related declines in balance, further underlining the clinical need for interventions that enhance vestibular function [10]. High fall-risk individuals identified through the Sensory Organization Test commonly displayed vestibular deficits [62]. This suggests that interventions like nEVS, which target vestibular system enhancement, may have the greatest impact on individuals with greater baseline impairments. Tailoring nEVS treatment based on individual vestibular thresholds could further optimize its effectiveness. While nEVS has previously been shown to induce short-term balance enhancements, the retention of these effects was limited [26]. However, the cumulative dosing treatments reported in the present study increased retention up to six months post-stimulation, suggesting neuroplastic changes and the potential to transition individuals from high- to low-fall risk profiles over 18 sessions.

The safety and tolerability of nEVS as an intervention is further supported by the absence of significant differences in headache frequency or MIDAS scores between the Stimulation and Sham groups, indicating that the intervention does not induce notable headaches or migraines. Both groups demonstrated significant improvements in balance confidence scores (PRE ABC vs. POST ABC), aligning with the reductions in Phybrata power and suggesting that nEVS not only enhances physical balance but also positively impacts participants’ confidence in their ability to perform balance-related tasks.

Pearson correlation analysis (Table 3) revealed few associations. In the Sham group, higher pre-intervention balance confidence was positively associated with lower baseline Phybrata power in the Floor EO condition and negatively associated in the Foam EO and Foam EC conditions. These mixed directions suggest that balance confidence may relate differently depending on the sensory demand of the condition. In the Stimulation group, a significant correlation between post-intervention Floor EO balance performance and SDMT scores, suggesting that better cognitive performance may relate to balance under simple sensory conditions. However, because Floor EO did not change significantly across sessions, these correlations should be interpreted with caution, as they may reflect stable individual differences rather than intervention-related effects. Overall, no consistent patterns were observed across other conditions or groups. As expected, older age was strongly associated with lower cognitive performance and reduced balance confidence, highlighting the multifaceted impact of aging on physical and psychological contributors to fall risk.

No significant relationships were observed between PASE scores and baseline or change in performance in either group across all conditions. This suggests that baseline physical activity levels did not predict the extent of improvement observed, reinforcing the accessibility of nEVS as a balance intervention that may be beneficial regardless of an individual’s physical activity. Although PASE did not significantly correlate with Phybrata power outcomes, future work should explore how long-term physical activity influences responsiveness to vestibular-based interventions.

Participants undergoing nEVS dosing can easily incorporate additional therapies tailored to their needs or combine them with balance and strength exercises to further enhance balance and mobility improvements. This versatility enhances nEVS both as a standalone treatment and as a complementary component of a holistic therapeutic strategy aimed at improving balance, mobility, and reducing fall risk in vulnerable populations. nEVS is easy to use, portable, low-cost, and minimally invasive, making it a potentially valuable tool to counteract age-related decline. Participants in this study reported no adverse side effects, aligning with findings from many other EVS studies [18, 23, 24, 63]. However, it is noteworthy that suprathreshold EVS (>1 mA) may induce more pronounced side effects, such as vertigo and metallic taste [64]. The portability of nEVS, combined with the head-mounted Phybrata sensor, facilitates seamless balance assessments and treatments, enabling integration into various settings, including retirement homes, for real-world data collection.

### Limitations

This study has several limitations. First, no a priori sample size calculation was performed, making it unclear whether the initial proof of concept study would be adequately powered to detect effects. However, this paper provides a foundation for sample size calculations to ensure that future studies are adequately powered. Effect sizes for the group variable were calculated using Cohen’s d in the LMM. Group had small-to-moderate effect sizes in Floor EC (d = 0.36), Foam EO (d = 0.39), and Foam EC (d = 0.32).

Second, while a single-blind design was implemented due to the subthreshold nature of the stimulation, participant blinding was only partially effective. Although participants were asked to guess their group allocation and the distribution of guesses was not significantly different from chance, 72% of the Stimulation group correctly identified their group, which raises concerns about placebo and expectancy effects. Thus, the Sham group served as a no-treatment control. This is particularly relevant given the late-emerging improvements in the Sham group (Foam EO and Foam EC conditions), which may reflect practice effects. Future studies should consider stimulation current ramping, intermittent placebo stimulation, or a double-blind study design to improve sham control.

Third, although the study excluded individuals with diagnosed vestibular dysfunction to isolate the effect of nEVS to normal aging populations, which may introduce ambiguity in interpreting the findings relative to vestibular pathology. The sample likely included older adults with presbyvestibulopathy, which could explain the observed improvements. However, the lack of direct vestibular function testing (e.g., vHIT, caloric, or VEMPs) limit the ability to characterize the vestibular profile of participants and related outcomes to specific deficits. Future studies should quantify vestibular function prior to, during, and following nEVS treatment to determine whether participants with vestibular hypofunction benefit to a greater degree from nEVS.

Fourth, our study protocol included fairly extensive balance testing before and after each stimulation session, so we effectively have studied the combination of nEVS and exercise-based treatment similar to Jain et al., with similar overall results [25]. Future studies may minimize the amount of additional balance testing to better isolate the balance improvements attributed to nEVS alone.

Fifth, the manual data cleaning process included removing artifacts of talking, head shaking, sneezing, or other anomalous movements which removed 1.94% of the total data. This introduces the potential for human error or subjective bias. Automation of this step using machine learning-based artifact detection could improve the reproducibility and reduce bias.

Sixth, the study’s generalizability is limited. The sample consisted of relatively healthy older adults. While participants with the poorest initial balance demonstrated the greatest improvements, the findings may not extend to populations with more sever balance impairments, neurological disorders, or confirmed vestibular deficits. Moreover, the effects of nEVS may vary across different stages of healthy aging and these differences remain unexplored. Future studies should examine how nEVS effects are modulated by age-related sensorimotor decline across the lifespan. However, the observed dose-response patterns suggest that individuals with greater impairments may benefit more, warranting larger sample sizes and targeted trials in clinical populations.

Finally, while the current study focused on standing balance, it did not assess gait which is critical to fall prevention and functional mobility. Future research should incorporate gait assessments, fall history, and quality of life measures to evaluate broader clinical outcomes and long-term impact of nEVS.

## Conclusion

This pilot study provides preliminary evidence that multi-session nEVS treatments may be a promising intervention for improving balance in older adults. We observed statistically significant improvements in postural control in several conditions, including those that increase reliance on vestibular inputs, such as Floor EC, Foam EO, and Foam EC. These finding suggest a potential benefit of nEVS for enhancing balance in cases where vestibular function plays a critical role. The results further suggest that nEVS may influence sensory reweighting and contribute to neuroplastic adaptations associated with balance control. Participants with lower baseline performance appeared to experience greater improvements, indicating that nEVS could have particular relevance for individuals at higher risk for balance impairments. These findings also support the hypothesis that nEVS may enhance both peripheral and central vestibular processing, contributing to improved postural stability. Additionally, the portability and scalability of nEVS make it an attractive option for both clinical and community use, from retirement homes to outpatient rehabilitation clinics to home care.

## Supplementary Information

Below is the link to the electronic supplementary material.

Supplementary Material 1: Table. Mean ± (SD) Phybrata power values for each group across sessions and conditions. This table reports mean ± standard deviation (SD) values of normalized Phybrata power for the Stimulation and Sham groups across four balance conditions: Floor Eyes Open (EO), Floor Eyes Closed (EC), Foam EO, and Foam EC. Data are shown for Sessions 1 through 18, as well as at 3-month (3 M) and 6-month (6 M) follow-ups. Session 1 values were normalized to 1.00; values for subsequent sessions reflect changes in performance relative to baseline. Lower values (i.e., negative deviations) indicate improved balance performance. Sample size (N) reflects the total number of participants per condition. The number of valid data points after outlier removal is indicated by n. All comparisons were conducted after removing extreme values defined by standard criteria. Significance was determined at the α = 0.05 level

## Data Availability

The dataset supporting the findings of this study is publicly available via Borealis: King, Jordan, 2025, Replication Data for: Electrical vestibular stimulation to improve static balance in older adults: a randomized control trial", 10.5683/SP3/SWWBUR, Borealis, V1.

## Abbreviations

ABC: Activities-specific balance confidence scale
CNS: Central nervous system
EO: Eyes open
EC: Eyes closed
EVS: Electrical vestibular stimulation
IMU: Inertial measurement unit
LMM: Linear mixed model
mCTSIB: Modified clinical test of sensory interaction on balance
MIDAS: Migraine disability assessment scale
nEVS: Noisy electrical vestibular stimulation
PASE: Physical activity scale for the elderly
SDMT: Symbol digit modalities test
VRT: Vestibular rehabilitation therapy

## References

[1] Public Health Agency of Canada. Seniors’ falls in Canada. Second report, Public Health Agency of Canada, Ottawa, ON, 2014. https://www.canada.ca/en/public-health/services/publications/healthy-living/seniors-falls-canada-second-report.html. Accessed 9 Aug 2024.

[2] Public Health Agency of Canada. Surveillance report on falls among older adults in Canada, Public Health Agency of Canada, Ottawa, Ontario, Canada, HP35-154/2022E-PDF, 2022.

[3] Iwasaki S, Yamasoba T. Dizziness and imbalance in the elderly: Age-related decline in the vestibular system. Aging Dis. 2015;6(1):38. 10.14336/AD.2014.0128. 25657851 10.14336/AD.2014.0128 PMC4306472

[4] Baloh RW, et al. Comparison of static and dynamic posturography in young and older normal people. J Am Geriatr Soc. 1994;42(4):405–12. 10.1111/j.1532-5415.1994.tb07489.x. 8144826 10.1111/j.1532-5415.1994.tb07489.x

[5] Parachute, cost of injury in Canada 2021. 2024. https://parachute.ca/en/professional-resource/cost-of-injury-in-canada/. Accessed 15 Jan 2025.

[6] World Health Organization. Ageing and health, 2024.

[7] Han BI, Song HS, Kim JS. Vestibular rehabilitation therapy: review of Indications, Mechanisms, and key exercises. J Clin Neurol. 2011;7(4):184. 10.3988/jcn.2011.7.4.184. 22259614 10.3988/jcn.2011.7.4.184 PMC3259492

[8] Brosel S, Strupp M. The vestibular system and ageing. In Harris JR, Korolchuk VI, editors. Biochemistry and cell biology of ageing: part II clinical science, in Subcellular Biochemistry, vol. 91. Singapore: Springer Singapore; 2019. pp. 195–225. 10.1007/978-981-13-3681-2_8 10.1007/978-981-13-3681-2_8 30888654

[9] Rossignol S, Dubuc R, Gossard J-P. Dynamic sensorimotor interactions in locomotion. Physiol Rev. 2006;86(1):89–154. 10.1152/physrev.00028.2005. 10.1152/physrev.00028.2005 16371596

[10] Zalewski C. Aging of the human vestibular system. Semin Hear. 2015;36(03):175–96. 10.1055/s-0035-1555120. 27516717 10.1055/s-0035-1555120 PMC4906308

[11] Agrawal Y, et al. Presbyvestibulopathy: diagnostic criteria consensus document of the classification committee of the Bárány society. J Vestib Res. 2019;29(4):161–70. 10.3233/VES-190672. 31306146 10.3233/VES-190672 PMC9249286

[12] Taylor RR, et al. Characterizing human vestibular sensory epithelia for experimental studies: new hair bundles on old tissue and implications for therapeutic interventions in ageing. Neurobiol Aging. 2015;36(6):2068–84. 10.1016/j.neurobiolaging.2015.02.013. 25818177 10.1016/j.neurobiolaging.2015.02.013 PMC4436436

[13] Jahn K. Inverse U-shaped curve for age dependency of torsional eye movement responses to galvanic vestibular stimulation. Brain. 2003;126(7):1579–89. 10.1093/brain/awg163. 12805121 10.1093/brain/awg163

[14] Dalton BH, Blouin J-S, Allen MD, Rice CL, Inglis JT. The altered vestibular-evoked myogenic and whole-body postural responses in old men during standing. Exp Gerontol, 60, pp. 120–8, 10.1016/j.exger.2014.09.020 10.1016/j.exger.2014.09.020 25456846

[15] Peters RM, Blouin J-S, Dalton BH, Inglis JT. Older adults demonstrate superior vestibular perception for virtual rotations. Exp Gerontol. 2016;82:50–7. 10.1016/j.exger.2016.05.014. 27262689 10.1016/j.exger.2016.05.014

[16] Baloh RW, Jacobson KM, Socotch TM. The effect of aging on visual-vestibuloocular responses. Exp Brain Res. 1993;95(3). 10.1007/BF00227144. 10.1007/BF00227144 8224077

[17] Haxby F, Akrami M, Zamani R. Finding a balance: a systematic review of the Biomechanical effects of vestibular prostheses on stability in humans. J Funct Morphol Kinesiol. 2020;5(2):23. 10.3390/jfmk5020023. 10.3390/jfmk5020023 PMC7739312 33467239

[18] Dlugaiczyk J, Gensberger KD, Straka H. Galvanic vestibular stimulation: from basic concepts to clinical applications. J Neurophysiol. 2019;121:2237–55. 10.1152/jn.00035.2019. 30995162 10.1152/jn.00035.2019

[19] Herssens N, McCrum C. Stimulating balance: recent advances in vestibular stimulation for balance and gait. J Neurophysiol. 2019;122(2):447–50. 10.1152/jn.00851.2018. 30864885 10.1152/jn.00851.2018

[20] White O, Babič J, Trenado C, Johannsen L, Goswami N. The promise of stochastic resonance in falls prevention. Front Physiol. 2019;9:1865. 10.3389/fphys.2018.01865. 30745883 10.3389/fphys.2018.01865 PMC6360177

[21] Lajoie K, Marigold DS, Valdés BA, Menon C. The potential of noisy galvanic vestibular stimulation for optimizing and assisting human performance. Neuropsychologia. 2021;152:107751. 10.1016/j.neuropsychologia.2021.107751. 33434573 10.1016/j.neuropsychologia.2021.107751

[22] Kwan A, Forbes PA, Mitchell DE, Blouin J-S, Cullen KE. Neural substrates, dynamics and thresholds of galvanic vestibular stimulation in the behaving primate. Nat Commun. 2019;10(1):1904. 10.1038/s41467-019-09738-1. 10.1038/s41467-019-09738-1 PMC6478681 31015434

[23] Lopez C, Cullen KE. Electrical stimulation of the peripheral and central vestibular system. Curr Opin Neurol. 2024;37(1):40–51. 10.1097/WCO.0000000000001228. 37889571 10.1097/WCO.0000000000001228 PMC10842451

[24] de Pires APB, Silva TR, Torres MS, Diniz ML, Tavares MC, Gonçalves DU. Galvanic vestibular stimulation and its applications: a systematic review. Braz J Otorhinolaryngol. 2022;88:S202–S211. 10.1016/j.bjorl.2022.05.010. 10.1016/j.bjorl.2022.05.010 PMC9760994 35915031

[25] Jain A, Sarkar A, Gupta M. Optimizing balance using vestibular electrical stimulation to study its therapeutic effect among elderly. Biosci Biotechnol Res Commun. 2022;15(2):313–20. 10.21786/bbrc/15.2.8.

[26] Fujimoto C, et al. Noisy galvanic vestibular stimulation induces a sustained improvement in body balance in elderly adults. Sci Rep. 2016;6(1):37575. 10.1038/srep37575. 27869225 10.1038/srep37575 PMC5116631

[27] Inukai Y, et al. Effect of noisy galvanic vestibular stimulation on center of pressure sway of static standing posture. Brain Stimulat. 2018;11(1):85–93. 10.1016/j.brs.2017.10.007. 10.1016/j.brs.2017.10.007 29079459

[28] McLaren R, Smith PF, Taylor RL, Niazi IK, Taylor D. Scoping out noisy galvanic vestibular stimulation: a review of the parameters used to improve postural control. Front Neurosci. 2023;17:1156796. 10.3389/fnins.2023.1156796. 37205050 10.3389/fnins.2023.1156796 PMC10187481

[29] Fu W, Bai Y, Wang X. Galvanic vestibular stimulation for postural rehabilitation in neurological disorders: a systematic review. Front Neurosci. 2025;19:1580078. 10.3389/fnins.2025.1580078. 40309657 10.3389/fnins.2025.1580078 PMC12040823

[30] Ahmed RAMA, Fahmy EM, Awad AM, Hamdy MM, Shaker HAAR. Efficacy of transmastoidal galvanic stimulation on recovery outcomes in patients with unilateral peripheral vestibular disorders: a randomized controlled trial. Egypt J Neurol Psychiatry Neurosurg. 2020;56(1). 10.1186/s41983-020-00207-x.

[31] Davis LE. Dizziness in elderly men. J Am Geriatr Soc. 1994;42(11):1184–8. 10.1111/j.1532-5415.1994.tb06986.x. 7963205 10.1111/j.1532-5415.1994.tb06986.x

[32] Agrawal Y. Aging, vestibular function, and balance: Proceedings of a National Institute on Aging/National Institute on Deafness and Other Communication Disorders Workshop. J Gerontol Ser A. 2020;75(12):2471–2480. 10.1093/gerona/glaa097. 10.1093/gerona/glaa097 PMC7662183 32617555

[33] Wagner AR, Akinsola O, Chaudhari AMW, Bigelow KE, Merfeld DM. Measuring vestibular contributions to Age-Related balance impairment: A review. Front Neurol. 2021;12:635305. 10.3389/fneur.2021.635305. 33633678 10.3389/fneur.2021.635305 PMC7900546

[34] Agrawal Y, Carey JP, Hoffman HJ, Sklare DA, Schubert MC, Adults US. Otol Neurotol. 2011;32(8):1309–11. 10.1097/MAO.0b013e31822e5bee. 21892121 10.1097/MAO.0b013e31822e5bee PMC3190311

[35] Cohen HS, et al. Screening for vestibular disorders using the modified clinical test of sensory interaction and balance and tandem walking with eyes closed. Otol Neurotol. 2019;40(5):658–65. 10.1097/MAO.0000000000002173. 31083095 10.1097/MAO.0000000000002173 PMC6530479

[36] Nguyen TT, Kang J-J, Oh S-Y. Thresholds for vestibular and cutaneous perception and oculomotor response induced by galvanic vestibular stimulation. Front Neurol. 2022;13:955088. 10.3389/fneur.2022.955088. 36034303 10.3389/fneur.2022.955088 PMC9413160

[37] Simoneau M, Nooristani M, Blouin J. Balance control threshold to vestibular stimuli. J Physiol. 2025;603(9):2783–99. 10.1113/JP288016. 40183736 10.1113/JP288016 PMC12072247

[38] Helmchen C, Rother M, Spliethoff P, Sprenger A. Increased brain responsivity to galvanic vestibular stimulation in bilateral vestibular failure. NeuroImage Clin. 2019;24:101942. 10.1016/j.nicl.2019.101942. 31382239 10.1016/j.nicl.2019.101942 PMC6690736

[39] Mitsutake T, Sakamoto M, Horikawa E. Comparing activated brain regions between noisy and conventional galvanic vestibular stimulation using functional magnetic resonance imaging. NeuroReport. 2021;32(7):583–7. 10.1097/WNR.0000000000001629. 33850089 10.1097/WNR.0000000000001629

[40] Forbes PA, Kwan A, Mitchell DE, Blouin J-S, Cullen KE. The neural basis for biased behavioral responses evoked by galvanic vestibular stimulation in primates. J Neurosci. 2023;43(11):1905–19. 10.1523/JNEUROSCI.0987-22.2023. 36732070 10.1523/JNEUROSCI.0987-22.2023 PMC10027042

[41] Ralston JD, Raina A, Benson BW, Peters RM, Roper JM, Ralston AB. Physiological vibration acceleration (Phybrata) sensor assessment of Multi-System physiological impairments and sensory reweighting following concussion. Med Devices Evid Res. 2020;13:411–38. 10.2147/MDER.S279521. 10.2147/MDER.S279521 PMC7733539 33324120

[42] Grafton ST, Ralston AB, Ralston JD. Monitoring of postural sway with a head-mounted wearable device: effects of gender, participant state, and concussion. Med Devices Evid Res. 2019;12:151–64. 10.2147/MDER.S205357. 10.2147/MDER.S205357 PMC6503189 31118838

[43] Abdollah V, Dief TN, Ralston J, Ho C, Rouhani H. Investigating the validity of a single tri-axial accelerometer mounted on the head for monitoring the activities of daily living and the timed-up and go test. Gait Posture. 2021;90:137–40. 10.1016/j.gaitpost.2021.08.020. 34481263 10.1016/j.gaitpost.2021.08.020

[44] Ralston J, Stanley S, Roper J, Ralston A. Phybrata digital biomarkers of Age-Related balance Impairments, sensory Reweighting, and intrinsic fall risk. Med Devices Evid Res. 2025;18:319–36. 10.2147/MDER.S522827. 10.2147/MDER.S522827 PMC12168941 40524937

[45] Lei Y, Rios V, Ji J, Duhon B, Boyd H, Xu Y. Quantifying unsupported sitting posture impairments in humans with cervical spinal cord injury using a head-mounted IMU sensor. Spinal Cord. 2024;62(2):65–70. 10.1038/s41393-023-00951-w. 38158410 10.1038/s41393-023-00951-w

[46] Filiatrault J, et al. Evidence of the psychometric qualities of a simplified version of the Activities-specific balance confidence scale for Community-Dwelling seniors. Arch Phys Med Rehabil. 2007;88(5):664–72. 10.1016/j.apmr.2007.02.003. 17466738 10.1016/j.apmr.2007.02.003

[47] Carone D, Sherman. A compendium of neuropsychological tests: fundamentals of neuropsychological assessment and test reviews for clinical practice (4th ed.). Appl Neuropsychol Adult. 2023;31:1–4. 10.1080/23279095.2023.2260143.

[48] Washburn RA, Smith KW, Jette AM, Janney CA. The physical activity scale for the elderly (PASE): development and evaluation. J Clin Epidemiol. 1993;46(2):153–62. 10.1016/0895-4356(93)90053-4. 8437031 10.1016/0895-4356(93)90053-4

[49] Stewart WF, et al. An international study to assess reliability of the migraine disability assessment (MIDAS) score. Neurology. 1999;53(5):988–988. 10.1212/WNL.53.5.988. 10496257 10.1212/wnl.53.5.988

[50] Grassi S, Frondaroli A, Pettorossi VE. Different metabotropic glutamate receptors play opposite roles in synaptic plasticity of the rat medial vestibular nuclei. J Physiol. 2002;543(3):795–806. 10.1113/jphysiol.2002.023424. 10.1113/jphysiol.2002.023424 PMC2290544 12231639

[51] Smith PF, Curthoys IS. Mechanisms of recovery following unilateral labyrinthectomy: a review. Brain Res Rev. 1989;14(2):155–80. 10.1016/0165-0173(89)90002-1. 2665890 10.1016/0165-0173(89)90013-1

[52] Dutia MB. Mechanisms of vestibular compensation: recent advances. Curr Opin Otolaryngol Head Neck Surg. 2010;18(5):420–4. 10.1097/MOO.0b013e32833de71f. 20693901 10.1097/MOO.0b013e32833de71f

[53] Kim G, Lee S, Kim K-S. Repeated galvanic vestibular stimulation modified the neuronal potential in the vestibular nucleus. Neural Plast. 2020;2020:1–14. 10.1155/2020/5743972. 10.1155/2020/5743972 PMC7273393 32565777

[54] Keywan A, Dietrich H, Wuehr M. Subliminal passive motion stimulation improves vestibular perception. Neuroscience. 2020;441:1–7. 10.1016/j.neuroscience.2020.05.053. 32505748 10.1016/j.neuroscience.2020.05.053

[55] Wagner AR, Sadeghi SG, Merfeld DM. Differential modification of perceptual thresholds by prolonged near threshold motion in healthy adults and after peripheral lesions. Front Neurol. 2025;16:1542496. 10.3389/fneur.2025.1542496. 40144627 10.3389/fneur.2025.1542496 PMC11936789

[56] Fitzpatrick RC, Watson SRD. Passive motion reduces vestibular balance and perceptual responses. J Physiol. 2015;593(10):2389–98. 10.1113/JP270334. 25809702 10.1113/JP270334 PMC4457199

[57] Alvarez JC, et al. Aging and the human vestibular nuclei: morphometric analysis. Mech Ageing Dev. 2000;114(3):149–72. 10.1016/S0047-6374(00)00098-1. 10802120 10.1016/s0047-6374(00)00098-1

[58] Goldberg JM, Smith CE, Fernandez C. Relation between discharge regularity and responses to externally applied galvanic currents in vestibular nerve afferents of the squirrel monkey. J Neurophysiol. 1984;51(6):1236–56. 10.1152/jn.1984.51.6.1236. 6737029 10.1152/jn.1984.51.6.1236

[59] Iwasaki S, et al. Noisy vestibular stimulation improves body balance in bilateral vestibulopathy. Neurology. 2014;82(11):969–75. 10.1212/WNL.0000000000000215. 24532279 10.1212/WNL.0000000000000215

[60] Gensberger KD, et al. Galvanic vestibular stimulation: cellular substrates and response patterns of neurons in the Vestibulo-Ocular network. J Neurosci. 2016;36:9097–110. 10.1523/JNEUROSCI.4239-15.2016. 27581452 10.1523/JNEUROSCI.4239-15.2016 PMC6601907

[61] Groves AK. The challenge of hair cell regeneration. Exp Biol Med Maywood NJ. 2010;235(4):434–46. 10.1258/ebm.2009.009281. 10.1258/ebm.2009.009281 PMC3773238 20407075

[62] Sung P, Rowland P. Impact of sensory reweighting strategies on postural control using the sensory organization test in older adults with and without fall risks. Physiother Res Int. 2024;29(2):e2075. 10.1002/pri.2075. 10.1002/pri.2075 38430540

[63] Utz KS, et al. Minor adverse effects of galvanic vestibular stimulation in persons with stroke and healthy individuals. Brain Inj. 2011;25(11):1058–69. 10.3109/02699052.2011.607789. 21879800 10.3109/02699052.2011.607789

[64] Cavin ID, Glover PM, Bowtell RW, Gowland PA. Thresholds for perceiving metallic taste at high magnetic field. J Magn Reson Imaging. 2007;26(5):1357–61. 10.1002/jmri.21153. 17969179 10.1002/jmri.21153

